# Failure to confirm influence of Methyltetrahydrofolate reductase (MTHFR) polymorphisms on age at onset of Huntington disease

**DOI:** 10.1186/1477-5751-4-12

**Published:** 2005-12-22

**Authors:** Wiebke Hansen, Carsten Saft, Jürgen Andrich, Thomas Müller, Stefan Wieczorek, Jörg T Epplen, Larissa Arning

**Affiliations:** 1 Department of Human Genetics, Ruhr-University, 44780 Bochum, Germany; 2 Department of Neurology, St. Josef-Hospital, Ruhr-University, 44791 Bochum, Germany

## Abstract

**Background:**

Huntington disease (HD) is a fully penetrant, autosomal dominantly inherited disorder associated with abnormal expansions of a stretch of perfect CAG repeats in the 5' part of the *IT15 *gene. The number of repeat units is highly predictive for the age at onset (AO) of the disorder. But AO is only modestly correlated with repeat length when intermediate HD expansions are considered. Recently, suggestive association has been reported between a single nucleotide polymorphism (SNP; *rs1801131*, also known as A1298C) in the methyltetrahydrofolate reductase (*MTHFR*) gene and AO of HD. 5,10-MTHFR is a key enzyme in the folate metabolism, diverting metabolites toward methylation reactions or nucleotide synthesis. Using part of a previously established study cohort plus additional patients and appropriate statistical methods, we reinvestigated two polymorphisms in the *MTHFR *gene, *C677T *and *A1298C*, as well as their association with AO in 167 HD patients.

**Results:**

There was no statistically significant impact on AO for HD patients, neither of *MTHFR *SNPs nor of the combinations thereof.

**Conclusion:**

Contrary to previously described evidence the A1298C polymorphism in the *MTHFR *gene does not appear to modulate AO of HD patients.

## Background

Huntington disease (HD) is caused by expansion of a cytosine-adenine-guanine (CAG) trinucleotide repeat in the 5'-translated region of the *IT15 *gene on chromosome 4, which encode the protein huntingtin [1]. The expansions result in the formation of elongated proteins with a variety of new properties. The extent of the expansion is inversely correlated with the age of onset (AO). Nevertheless, large part of the variance in AO remains unexplained [2]. The pathogenesis of HD has been implicated to relate to different aspects of the homocysteine metabolism: Cystathionine [beta]-synthase (CBS) appears to bind specifically to huntingtin (htt) [3]. CBS deficiency is associated with homocystinuria, which affects various physiological systems, including the central nervous system. Homocysteine, one of the substrates of CBS accumulates in homocystinuria and is metabolized to homocysteate and homocysteine sulphinate, both components of which are amino acids with significant excitotoxic potential. In this context homocysteine was suggested to influence the pathogenesis of HD. Two common polymorphisms have been described in the *MTHFR *gene, both single nucleotide substitutions resulting in amino acid changes (*C677T *→ *Ala222Val *and *A1298C *→ *Glu429Ala*) [4,5]. Whereas *C677T *unequivocally affects enzyme function and has been associated with increased plasma homocysteine concentrations and an altered balance of folate metabolites [4], the functional relevance *in vivo *of the *A1298C *allele is less well defined. *A1298C *affects enzyme function *in vitro *to a lesser degree, and individuals carrying the variation have frequently normal homocysteine and plasma folate concentrations [6,7]. It is unclear whether the substitution affects folate metabolism under specific physiological conditions, *e.g*. under low nutrient intake. Apparently, the 677C→T and the 1298A→C polymorphisms can act synergistically, given that heterozygosity for both polymorphisms causes lower MTHFR enzymatic activity than heterozygosity alone for either of them and a trend to higher or significantly higher plasma total homocysteine levels [8].

Brune et al. reported recently an association between the homozygous A1298C allele and a distinctly lower AO compared to the wild type *MTHFR *genotypes [9]. In the present study we re-examined the 1298A→C polymorphism as well as its potential interaction with the 677C→T polymorphism as genetic factors influencing the AO of HD. Compared to the patient cohort examined by Brune et al. (n = 171), here 27 patients have been excluded from the initial cohort due to relatedness (the first diagnosed family member remained in this study) and lacking information on the motor age at onset. In contrast to the initial cohort, exclusively the motor AO was referred to. The present cohort (n = 167) has been supplemented by 23 patients due to recruitment of new patients. The potential influence of certain genotypes on AO was calculated by linear regression, in which R^2 ^illustrates the relative improvement of the regression model when the various genotypes are considered in addition to the HD CAG repeats.

## Results and discussion

Analysis of the *MTHFR *677C→T and the 1298A→C polymorphisms in 167 patients revealed allele frequencies of 0.35 for *MTHFR *677T and 0.29 for *MTHFR *1298C, respectively. Observed frequencies were in Hardy-Weinberg equilibrium.

The prevalences of the combined *MTHFR *genotypes for patients and controls are listed in Table 1. 23.3% of the subjects represented combined heterozygotes for the two SNPs (1298AC/677CT). We found no double homozygous individuals (1298CC/677TT) and no patient carrying the 1298CC/677CT genotype, a result to be expected based on genotype frequencies reported in other populations [11,12]. Thus our findings comply with the suspicion that these two polymorphisms occur rarely in *cis *[7]. Addition of the *MTHFR *genotype variations, alone and in combination (data only shown for the dominant model of the rare allele or the model for compound heterozygosity, respectively) to the effect of CAG repeat lengths resulted in no significant increase in the R^2 ^value (Table 2). Hence, this study failed to replicate the association finding between the genotypes of the A1298C polymorphism in *MTHFR *with the AO of HD. Since our cohort comprises mostly the same individuals as investigated before (144/167), the initial description of association is due to weaker exclusion criteria concerning relatedness of patients as well as exclusive reference to motor AO. In addition different statistical principles were employed.

**Table 1:** Number of different genotype combinations of the *MTHFR *677C→T^a ^and 1298 A→C^b ^polymorphism in 167 HD patients

***MTHFR***	**677CC (%)**	**677CT (%)**	**677TT (%)**
**genotype**	**n = 71**	**n = 75**	**n = 21**
**1298AA (n = 80)**	23 (13.7)	37 (22.1)	20 (12)
**1298AC (n = 74)**	35 (21)	38 (22.8)	1 (0.6)
**1298CC(n = 13)**	13(7.8)	0	0

^a^Allele frequency of *MTHFR *677T amounts to 0.35.

^b^Allele frequency of *MTHFR *1298C amounts to 0.29.

**Table 2:** Linear regression analysis concerning polymorphisms in the *MTHFR *gene

**Gene (polymorphism)**	**R**^2^	Δ**R^2^**	**% additional explained variance**	**P value**
HD CAG	.308	-		< .0005

HD CAG + *MTHFR *A1298C	.304	.004	-	.850
HD CAG + *MTHFR C677T*	.305	.003	-	.590
HD CAG + *compound heterozygotes*	.304	.004	-	.828

Variance in age at onset for the CAG repeats is indicated as such as well as in combination with the different polymorphisms examined. R^2 ^illustrates the relative improvement of the regression model when the various genotypes are considered in addition to the HD CAG repeats, ΔR^2 ^values quantify these differences. P values refer to R^2^.

## Conclusion

We failed to replicate the association finding between the *1298CC *genotype in the *MTHFR *gene and earlier AO in HD. In future studies in this context, also the folate levels of individual patients should be taken into account as well as environmental factors.

## Methods

One hundred sixty-seven patients clinically diagnosed as suffering from HD were ascertained for their motor AO in the Huntington Center (HZ) NRW, Bochum (Germany) [10]. All patients gave informed consent for genotyping. The CAG repeat sizes and the *MTHFR *A1298C and *C677T *genotypes were determined as described before [9]. The dependence of the AO on CAG repeat number was determined by linear regression. Residuals from this model were verified, and there was no evidence of departure from normality and equality of variance assumptions. The possible genotypic effects of the two polymorphisms were assessed with multiple linear regressions, while allowing for the predictive effects of the CAG repeat size. We used the AO as dependent variable and the respective genotypes as independent variables. The CAG repeat number was considered as numerical variable. All of the other putatively modifying genotypes were considered as nominal variables by assigning the value "0, 1" or "0, 1, 2" according to the subject's number of variant alleles under a model of dominance or otherwise according to a model of generalized additive allelic effect. SPSS Ver.11.0 for Windows (SPSS Inc.) was used for all statistical analyses.

## Competing interests

The author(s) declare that they have no competing interests.

## Authors' contributions

WH and LA initiated the study; WH carried out the molecular genetic studies and drafted the manuscript. JA and CS had ascertained the clinical status of the patients. SW and JTE participated in the study design and finalized the analyses as well as several versions of the paper.

All authors read and approved the final version of the manuscript.
